# A case of Henoch–Schönlein purpura with underlying TRAPS (tumour necrosis factor receptor-associated periodic syndrome)

**DOI:** 10.1093/rap/rkac077

**Published:** 2022-09-10

**Authors:** Kanishk Jain, Anurag Bharadwaj

**Affiliations:** Department of Rheumatology, Mid & South Essex Foundation Trust, Basildon University Hospital, Basildon, UK; Department of Rheumatology, Mid & South Essex Foundation Trust, Basildon University Hospital, Basildon, UK

Key message

- Consider alternative diagnosis with recurrent attacks of Henoch–Schönlein purpura and partial response to CSs.

Dear Editor, A 22-year-old Caucasian man with no previously diagnosed illness presented to the emergency department with a 1-week history of bilateral lower limb rash. Past history was pertinent for recurrent non-specific childhood abdominal pain persisting into early adulthood. The rash was non-blanching, purpuric, palpable and maculopapular in nature, extending up to the knee, with associated arthralgia (Fig. 1). Routine blood tests revealed neutrophilia, raised inflammatory markers, elevated serum IgA with preserved renal function. Florid leucocytoclastic vasculitis affecting dermal blood vessels with prominent neutrophilic micro-abscesses was evident on skin biopsy. Immunofluorescence was notable for moderate IgA staining of dermal vessels along with fibrinogen. In view of the characteristic joint and skin findings with elevated serum IgA, a diagnosis of Henoch–Schönlein purpura (HSP; IgA vasculitis) was firmly established.

**Figure 1.**
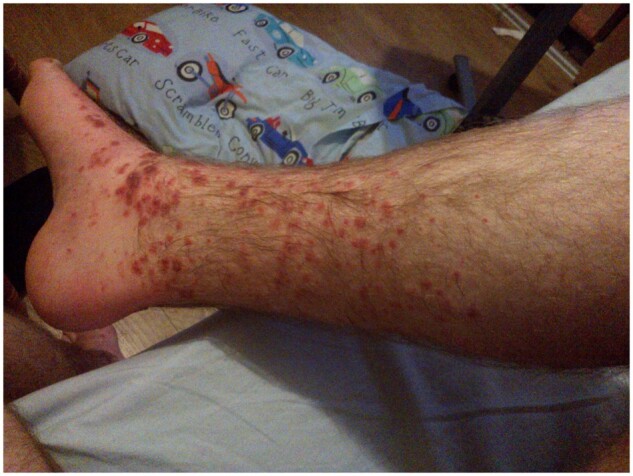
Vasculitic rash affecting ankle and shin at initial presentation

The skin rash and arthralgia promptly dissipated on initiation of topical CSs. However, inflammatory markers were less responsive, and he developed persistent mild proteinuria and microscopic haematuria requiring multiple courses of oral CS therapy. Over a course of 5 years, he had a total of seven inpatient stays with relapse of rash on CS taper, recurrent abdominal pain and persistently raised CRP. In each instance, he was treated as a flare-up of his HSP. Several incidental findings accumulated, including low-grade colitis, gastritis, cryofibrinogenaemia, hypoalbuminaemia and microcytic anaemia. These features were thought to be consistent with underlying autoimmune vasculitis. Eventually, declining CS responsiveness culminated in acute presentation of worsening abdominal pain, moderate reactive ascites, limb swelling and pleuropericardial effusion. The possibility of a multisystem autoinflammatory disorder was entertained. Genetic testing conducted at a specialist unit confirmed him as a *TNFRSF1A* Tyr38Cys (Y38C) heterozygote, and a diagnosis of tumour necrosis factor receptor-associated periodic syndrome (TRAPS) was established. He subsequently began treatment with the IL-1 antagonist anakinra. Marked and sustained improvement was noted in clinical and inflammatory parameters, with no further hospital admission or CS requirement.

In retrospect, the episodes of non-specific childhood abdominal pain were likely to be related to repeated TRAPS flare-up. A total of 10 documented contacts with medical care were made, with presumptive diagnoses of dairy allergy, abdominal migraine, psychogenic abdominal pain, gastroenteritis and anxiety attack. At the time of presentation, a skin biopsy revealed features of leucocytoclastic vasculitis and IgA staining. Although several differentials for leucocytoclastic vasculitis exist, the presence of IgA deposits is highly suggestive of HSP [1]. Although cutaneous manifestations are also common in TRAPS, they tend to present as migratory erythema with dermal infiltration from T cells and monocytes [2], as opposed to the neutrophilic infiltrates seen in this case. The HSP course was atypical owing to resistant vasculitic rash and inflammatory markers. An isolated cryofibrinogen band was detected on two separate occasions with no cryoprecipitate; this was deemed to be of unclear significance. A historical case report has documented presence of cryofibrinogen during the active HSP phase [3]. Moreover, cryofibrinogenaemic vasculitis could be a reasonable differential. Isolated cryofibrinogenaemia is commonly associated with cutaneous purpura, leucocytoclastic vasculitis and fibrinogen in dermal vessels, as seen in this case. However, there are no well-accepted criteria for cryofibrinogenaemia, and it was deemed less likely in view of positive IgA staining, absence of cold sensitivity and vasocclusive disease. Eventually, on developing worsening abdominal pain, unexplained ascites, limb swelling and serositis, a diagnosis of underlying autoinflammatory disease was considered. Retrospectively, he fulfilled the Eurofever proposed diagnostic criteria for TRAPS [4] based on the following: episode durations >6 days, migratory erythematous patches, myalgia, and absence of vomiting or aphthous stomatitis. TRAPS is a rare autosomal-dominant periodic syndrome resulting from missense mutation in the TNF1 receptor. Characteristic features include long-lasting febrile episodes, limb pain, abdominal pain, ocular inflammation, serositis and rash. Of note, prolonged fevers and associated family history were not prominent features in his presentation.

This case follows a unique course, with a biopsy-confirmed diagnosis of adult-onset HSP later revealing underlying TRAPS on genetic testing. The rarity of co-existing vasculitis and autoinflammatory syndrome might suggest co-incidental causation, but an exaggerated innate immune response may explain the interlink [5]. HSP has been documented in ≤7% of patients with FMF [6], while its link with TRAPS is less well established. Genetic studies have implicated TNF signalling defects in several autoimmune diseases, including Crohn’s disease, type I diabetes and SS [7]. In particular, *TNFRSF1A* mutation has also been associated with the development of multiple sclerosis [8].

In conclusion, we highlight that both conditions can present in adulthood and, furthermore, co-exist, with the underlying immunogenic link leading to repeated attacks of vasculitis unmasking florid manifestations of autoinflammatory syndrome. The possibility of an autoinflammatory syndrome should be considered in the setting of unexplained, episodic or persistent multi-system inflammation, repeated hospital admissions and recurrent abdominal or limb pain. Furthermore, an alternative diagnosis should be evaluated in cases of recurrent HSP with partial response to CSs.

## Data Availability

There are no relevant data other than the confidential patient file.
